# Early nanoparticle intervention preserves motor function following cervical spinal cord injury

**DOI:** 10.1002/btm2.70011

**Published:** 2025-04-29

**Authors:** Sarah E. Hocevar, Brian C. Ross, Yinghao Wang, Cecelia R. Crowther, Samantha R. Schwartz, Brain J. Cummings, Aileen J. Anderson, Lonnie D. Shea

**Affiliations:** ^1^ Neuroscience Graduate Program University of Michigan Medical School Ann Arbor Michigan USA; ^2^ Department of Biomedical Engineering University of Michigan Ann Arbor Michigan USA; ^3^ Institute for Memory Impairments and Neurological Disorders University of California, Biological Sciences III Irvine California USA; ^4^ Sue and Bill Gross Stem Cell Research Center University of California Irvine California USA; ^5^ Physical Medicine and Rehabilitation University of California Irvine California USA; ^6^ Department of Chemical Engineering University of Michigan Ann Arbor Michigan USA

**Keywords:** immunomodulation, inflammatory response, nanoparticle, spinal cord injury

## Abstract

Spinal cord injury (SCI) triggers an immediate influx of immune cells that secrete pro‐inflammatory cytokines and reactive oxygen species that cause tissue damage that is secondary to the initial physical trauma. We aim to reprogram these immune cells to promote a less inflammatory and more pro‐regenerative environment. Herein, we investigated the window in time during which poly(lactide‐co‐glycolide) nanoparticles (NPs) administration can successfully modulate the immune response and promote functional sparing. The dynamics of immune cell infiltration and secondary tissue damage were studied following the injection of NPs intravenously every 24 h for 7 days following injury, with the first injection starting at 2, 4, or 24 hours post‐injury (hpi). At 7 days post‐injury (dpi), early NP intervention decreased the number of infiltrating macrophages and neutrophils, but delaying treatment until 24 hpi increased the number of neutrophils above control. All mice that received NPs had greater neuronal sparing contralateral to the injury, but mice that received NPs at early timepoints had greater neuromuscular junction innervation and motor endplate sparing. The increased sparing of neurons and neural circuits in the 2 hpi NP group corresponded with increased motor function, as measured by a ladder beam test. Collectively, these results suggest that early intervention with NPs can modulate the inflammatory response and preserve motor function and circuits following SCI.

**Translational Impact Statement**

Inflammation following spinal cord injury causes secondary damage to neural tissue and exacerbates functional loss. There are currently no standard‐of‐care treatments that address inflammation after spinal cord injury. This study describes the use of drug‐free, biodegradable poly(lactide‐co‐glycolide) nanoparticles to acutely reprogram peripheral immune cells following spinal cord injury (SCI), which can reduce inflammation and the associated damage and lead to improved functional recovery.

## 1 INTRODUCTION

The United States alone has approximately 17,000 new cases of spinal cord injury (SCI) each year.^1^ Following injury, circulating neutrophils infiltrate the spinal cord within hours of injury and release inflammatory cytokines and reactive oxygen and nitrogen species at the injury site that exacerbate neuronal and oligodendrocyte cell death.^2^, ^3^, ^4^ Methylprednisolone, a glucocorticoid that downregulates adhesion molecule and cytokine production in immune cells, has been investigated in clinical trials to decrease the inflammatory response to SCI.^5^, ^6^, ^7^ However, while some patients exhibited improved motor and sensory function, its use has been discontinued because of its many off‐target effects including sepsis, pneumonia, impaired wound healing, and gastrointestinal bleeding. Additionally, mouse studies have shown that complete ablation of neutrophils and macrophages does not significantly improve motor performance and has a negative effect on wound healing and regrowth into the injury.^8^, ^9^, ^10^ Together, these studies demonstrate the need for a clinical intervention that can attenuate immune cells during spinal cord injury without causing widespread immunosuppression and its negative effects.

An emerging strategy for SCI to address inflammation is polarizing innate immune cells toward a less‐inflammatory phenotype, which can lead to phenotypes that promote rather than diminish functional recovery. Biomaterial technologies, in particular, have emerged as tools to locally modulate inflammation and enhance the regenerative potential of the spinal cord. Recently, nanoparticles (NPs) have been reported to target spinal cord injury due to their ability to cross the semipermeable blood–brain barrier.^11^, ^12^ Nanoparticles are often conjugated to a therapeutic agent and administered systemically, yet limited accumulation of NPs at the injury limits the extent of the therapeutic effect. Conjugating additional guiding peptides onto the NPs can increase accumulation in the spinal cord, but the therapeutic effect can still be limited by the low phagocytic ability of many resident cells of the spinal cord.^13^, ^14^, ^15^, ^16^ To circumvent these accumulation issues and the off‐target effects of drugs like methylprednisolone, our lab has previously developed cargo‐less poly(lactide‐co‐glycolide) (PLG) NPs that are phagocytosed by circulating myeloid cells and reprogram them toward a more anti‐inflammatory phenotype. Administration of these NPs led to increased axon regrowth into the injury and improved functional recovery in mice as early as 7 days post‐injury that were sustained for at least 12 weeks.^17^

In this report, we investigate the cellular processes that are influenced by NP administration, with assessment of the immune dynamics following administration and sparing of neural tissue. The studies employ a cervical hemisection injury, and the timing of initial NP administration was varied between 2 and 24 h, with daily NP injections subsequently for 1 week. This model was chosen instead of the more common contusion model due to more severe functional and behavioral outcomes following penetrating injuries. The varied timing of first NP administration was anticipated to target different populations of infiltrating immune cells. An administration time of 2 h would target neutrophil and monocyte populations that are upregulated following injury, whereas a 4‐h time point would preferentially target monocytes.^18^, ^19^ An administration time of 24 h was chosen because both neutrophils and monocytes have already begun to accumulate in the injury.^20^ Because this study is a continuation of our previous work, we elected to use the same injury model, which includes the implantation of a biomaterial bridge that limits scar formation and supports axon growth into the injury. The presence of the bridge, however, increases the permeability of the injury, and NPs can accumulate in the injury without being transported by immune cells.^21^ While the numbers of infiltrating immune cells are similar in NP‐treated and control mice 2 days post‐injury, fewer cells were present by the end of the first week post‐injury. All NP conditions led to increased survival of neurons in the spinal cord compared to controls, but earlier intervention improved neuromuscular junction innervation (NMJ) and motor performance. Collectively, these results support the use of NPs as a means to modulate the acute inflammatory response to improve outcomes in spinal cord injury.

## 2 METHODS

### 2.1 PLG NP fabrication and administration

PLG NPs were made by dissolving 50:50 PLG (inherent viscosity = 0.55 dL/g; Evonik, Sand Creek, MI) in dichloromethane along with poly(ethylene‐alt‐maleic anhydride) (Polysciences, Inc., Warrington, PA). The mixture was sonicated with a Cole‐Parmer CPX130 Ultrasonic Processor (Vernon Hills, IL) and then stirred overnight to evaporate the organic phase. The following day, the NPs were collected and washed with water to produce particles that are 626.95 ± 47.67 nm in size (average pdi = 0.19) and −47.86 ± 4.38 mV in charge. Particle size and charge were measured with a Zetasizer Nano ZSP (Malvern Panalytical, Malvern, UK). The particles were lyophilized with sucrose and D‐mannitol (Sigma‐Aldrich, St. Louis, MO). During administration, each mouse received 0.1 mL of 10 mg/mL NPs in DPBS injected into the lateral tail vein daily for 1 week following injury. Control mice received 0.1 mL DPBS instead of NPs. The timing of the first NP injection varied from 2 hours post‐injury (hpi) up to 24 hpi. Every subsequent NP injection was 24 h after the previous injection so that NP injections remained staggered between different timing conditions.

### 2.2 Multichannel PLG bridge fabrication

Bridges were fabricated using the same protocol previously published by our lab.^22^, ^23^, ^24^ PLG (75:25 lactide:glycolide; inherent viscosity = 0.76 dL/g; Lakeshore Biomaterials, Birmingham, AL) was dissolved in dichloromethane (6% w/w) and emulsified in 1% poly (vinyl‐alcohol) (Millipore Sigma, Burlington, MA) using a homogenizer (PolyTron 3100; Kinematica AG, Littau, Switzerland). The resulting microspheres were approximately 1 μm in diameter. D‐sucrose (Millipore Sigma), D‐glucose (Millipore Sigma), and dextran MW 100,000 (Millipore Sigma) were mixed at a ratio of 5.3:2.5:1, respectively, by mass and then were caramelized, cooled, and drawn from solution using a Pasteur pipette to create sugar strands. Each 150–250 μm strand was coated with a 1:1 mixture of PLG microspheres and salt (63–106 mm) and pressed into a salt‐lined aluminum mold. The strands were used to create channels to act as a conduit for regenerating axon bundles, and salt was used to create a microporous structure. The materials were gas‐foamed under 800 psi for 16 h to fuse the particles into a matrix. The bridges were cut into 1.15 mm long pieces, and the NaCl was leached with water, leaving behind a multichannel bridge.

### 2.3 Spinal cord hemisection model and animal care

All animal work was performed according to protocol number PRO00011456 approved by the Institutional Animal Care and Use Committee (IACUC) at the University of Michigan. A C5 lateral hemisection was performed on 6–8‐week‐old male and female C57BL/6J (Jackson Laboratory, Bar Harbor, ME) or Crym‐RFP (cross between Mutant Mouse Resource & Research Center #036627‐UCD and Jackson Laboratory #007905) mice as previously described.^22^ C57BL/6J mice were only used in flow cytometry experiments; all behavioral and histology experiments used Crym‐RFP mice that have been used in our previous studies. Mice were anesthetized with 2% isoflurane, and 1 mg/kg bupivacaine was administered as a local anesthetic. After a C5 laminectomy was performed, a 1.15 mm lateral hemisection of the left side of the spinal cord was removed to allow for PLG bridge implantation. The injury and exposed spinal cord were covered in Gelfoam (Ethicon, Raritan, NJ). Muscles were sutured using 5/0 Chromic Gut (Henry Schein, Melville, NY), and the skin was stapled. Post‐operative care consisted of buprenorphine (0.1 mg/kg) twice daily for 3 days, enrofloxacin (2.5 mg/kg, Bayer, Leverkusen, Germany) daily for 14 days, and lactated Ringer's solution (1 mL, MWI Veterinary Supply, Boise, ID) daily for 5 days. Bladders were expressed twice daily until function recovered, and wound clips were removed 10 days after surgery.

### 2.4 Behavioral analysis

The horizontal ladder beam test was used to evaluate locomotor function over a period of 12 weeks following injury. Mice were trained on the ladder beam consisting of 50 rungs during the week prior to injury. Mice were recorded at 1, 2, and every 2 weeks up to 12 weeks following injury. Videos were scored by two blinded observers for 3 trials per animal by counting the number of correct and misplaced steps of the left forepaw on the ladder beam.

### 2.5 Immunohistochemistry

At 1 week post‐injury, mice were euthanized with 5% isoflurane, and C4–C6, along with the left acromiotrapezius and forearm flexor muscles, were dissected. Tissues were flash frozen in isopentane on dry ice and embedded in 20% w/w sucrose (Millipore Sigma) in OCT (ThermoFisher Scientific). Muscles and spinal cords were cryosectioned at 14 μm thick longitudinally and transversely, respectively. Tissue sections were permeabilized in 0.5% Triton in Tris‐buffered saline, fixed with 4% paraformaldehyde, and blocked with normal goat or normal donkey serum as necessary.

Antibodies used were mouse anti‐NeuN (1:250, Millipore, Burlington, MA, #MAB377) for mature neurons, chicken anti‐NF200 (1:250, AVES Labs, Davis, CA, #NFH) for neurofilaments, anti‐bungarotoxin conjugated to Alexa Fluor 647 (1:500, ThermoFisher, Waltham, MA, #B35450) for motor endplates, rat anti‐F4/80 (1:200, Bio‐rad MCA497GA) for macrophages, and rat anti‐Ly6G (Biolegend #127602) for neutrophils. Secondary antibodies were AlexaFluor 555 goat anti‐rat (1:1000; Invitrogen #A21434), AlexaFluor 555 goat anti‐mouse (1:1000; Invitrogen #A21424), and AlexaFluor 488 goat anti‐chicken (1:1000; Invitrogen #A11039). Tissues were imaged on an Axio Observer Z1 using an ORCA‐Flash 4.0V2 Digital CMOS camera.

### 2.6 Quantification of IHC

For immune cell and neuron IHC, two slides with four tissue sections were used per mouse. Tissue sections spanned the length of the C5 injury. Tissue sections above or below the level of injury were not used. For NMJ IHC, two slides with six tissue sections spanning the depth of the muscle were used per mouse. To quantify immune cells in the spinal cord, images were exported as .CZI files and converted to .H5 files in CellProfiler.^25^ The injury area was outlined using the polygon tool in Fiji and saved as a mask.^26^ The .H5 files were uploaded to Ilastik, and pixel classification was performed after training a set of 4 randomly selected tissues per condition.^27^ A single operator trained Ilastik to ensure consistent staining identification, and the program was trained against a no‐primary control to prevent identification of non‐specific staining. Immune cells were identified by colocalization of greater than three pixels of either F4/80 for macrophages/microglia or Ly6G for neutrophils with DAPI. Only immune cells in the injury area were counted.

The number of NeuN+ neurons on the C5 uninjured side of the spinal cord contralateral to the injury was manually counted by two independent, blinded observers using Fiji. BGT+ motor endplates and innervated motor endplates, defined as colocalization of NFM+ axons with BGT+ motor endplates, were manually counted by two independent, blinded observers using Fiji. The entire longitudinal muscle section was quantified using a total of 12 tissue sections per mouse. The polygon tool in Fiji was used to outline the muscle section and measure the area to determine NMJ density.

### 2.7 Flow cytometry

At 2, 5, or 7 dpi, mice were euthanized with 5% isoflurane, and C4–C6 of the spinal cord was dissected, including both the injury site and contralateral side. For uninjured controls, C4–C6 was also dissected. Trypsin (0.5 mg; Millipore Sigma) and collagenase (1 mg; Millipore Sigma) in DMEM were added to a tube with the spinal cords, and the spinal cords were minced with microscissors. Following mincing, tissues were incubated at 37°C in a rotisserie shaker for 20 min. The samples were spun down in a microcentrifuge and neutralized with DMEM with 10% FBS (ThermoFisher Scientific). The samples were spun down again, and supernatant was aspirated. Then, the tissues were myelin depleted using the Miltenyi Myelin Removal Beads II kit (Bergisch Gladbach, Germany). Following myelin depletion, cells were counted, and 1 million cells per animal were used for flow cytometry. Fixable Live/Dead Violet was used to assess cell viability. Samples were incubated with anti‐CD16/32 (1:100, clone 93; Biolegend) to block nonspecific staining. Cells were additionally incubated with antibodies for CD11b BV421 (0.2 μg/μL; Biolegend 101251) Ly6C BV510 (0.1 μg/μL; Biolegend 128033), Ly6G PE (0.2 μg/μL; Biolegend 127608), F4/80 AF647 (0.5 μg/μL; Biolegend 157314), and CD45 FITC (0.5 μg/μL; Biolegend 103108) and analyzed using a BioRad ZE5 Cell Analyzer. Data analysis was performed using FlowJo (BD).

### 2.8 Statistical analysis

Statistical analysis was performed in Graphpad Prism 10. All values are expressed as the mean ± standard error of the mean. For flow cytometry, *n* = 5–6 mice were used. For immunohistological data, *n* = 4–10 mice were used. The number of mice used in a specific figure is noted in the figure caption. For flow cytometry and immunohistochemistry, an ordinary one‐way ANOVA was used with Tukey's post hoc test to correct for multiple comparisons. For ladder beam data, *n* = 10 mice were used and a two‐way ANOVA with Tukey's post hoc test was performed. The *p*‐values for statistical significance are represented with stars (unless otherwise noted: *p < 0.05, **p < 0.005, ***p < 0.0005, ****p < 0.0001). While both male and female mice were used, studies were not sufficiently powered to analyze sex differences.

## 3 RESULTS

### 3.1 Immune cell dynamics across the first week of injury

**FIGURE 1:**
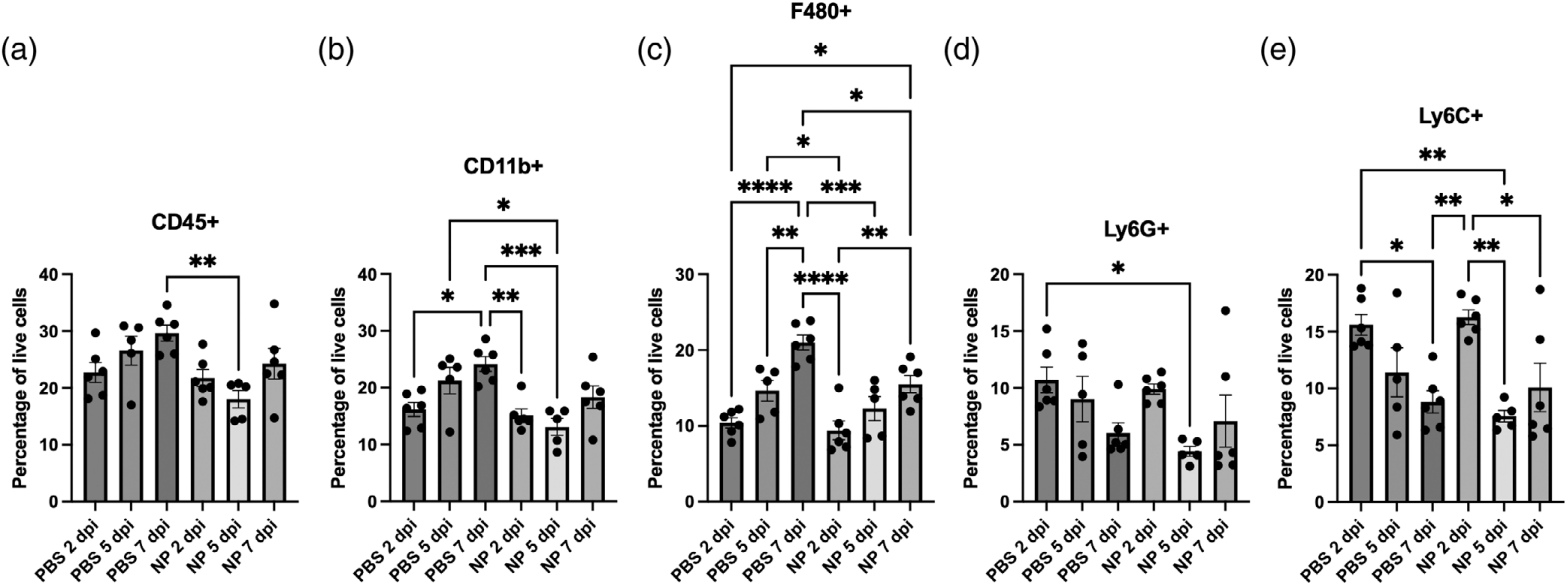
Immune cell population dynamics in spinal cord across the first week of injury. Mice were given daily injections of NPs or PBS starting at 2 hpi. Spinal cords were harvested at multiple timepoints across the first week of injury. Spinal cords were disassociated and myelin depleted before flow cytometry for immune cell surface markers for total immune cells (a), myeloid cells (b), macrophages and microglia (c), neutrophils (d), and monocytes (e). Data are mean ± SEM. *n* = 5–6 mice per condition. *p < 0.05, **p < 0.01, ***p < 0.001, ****p < 0.0001.

We first analyzed the immune cell response in the spinal cord throughout the first week of injury with and without NP intervention. Our previous study using NPs in a hemisection model of SCI showed differences in immune cell accumulation at the injury at 1 week post‐injury; therefore, we wanted to further explore the inflammation dynamics at earlier timepoints in the first week of injury.^17^ Administered NPs were 626.95 ± 47.67 nm in size (average pdi = 0.19) and −47.86 ± 4.38 mV in charge, which were similar in size and charge to NPs used in previous studies by our lab.^17^, ^28^ Mice received NPs or PBS starting within 2 hpi and received daily NP injections. Flow cytometry for immune cells was performed at 2, 5, or 7 days post‐injury (dpi). NP administration trended toward a lower percentage of total CD45+ cells in the spinal cord compared to the PBS control at 5 dpi (*p* = 0.0701) (Figure 1a). The percentage of CD11b+ myeloid cells significantly increased across the first week for PBS‐treated mice (*p* = 0.0116), yet not for NP‐treated mice (Figure 1b). At 5 dpi, the percentage of myeloid cells was significantly lower in mice that received NPs (*p* = 0.0222). Within each condition, the percentage of F4/80+ cells (macrophages and microglia) increased across the week, and by Day 7, significantly fewer F4/80+ cells were observed in mice that received NPs (*p* = 0.0207) (Figure 1c). The percentages of Ly6G+ neutrophils (Figure 1d) and Ly6C+ monocytes (Figure 1e) decreased across the first week within each condition and at Day 5 were lower in NP‐treated mice. In total, these results show that NPs are able to decrease the accumulation of innate immune cells during the first week after injury.

### 3.2 Nanoparticle administration alters immune infiltration into the spinal cord

**FIGURE 2:**
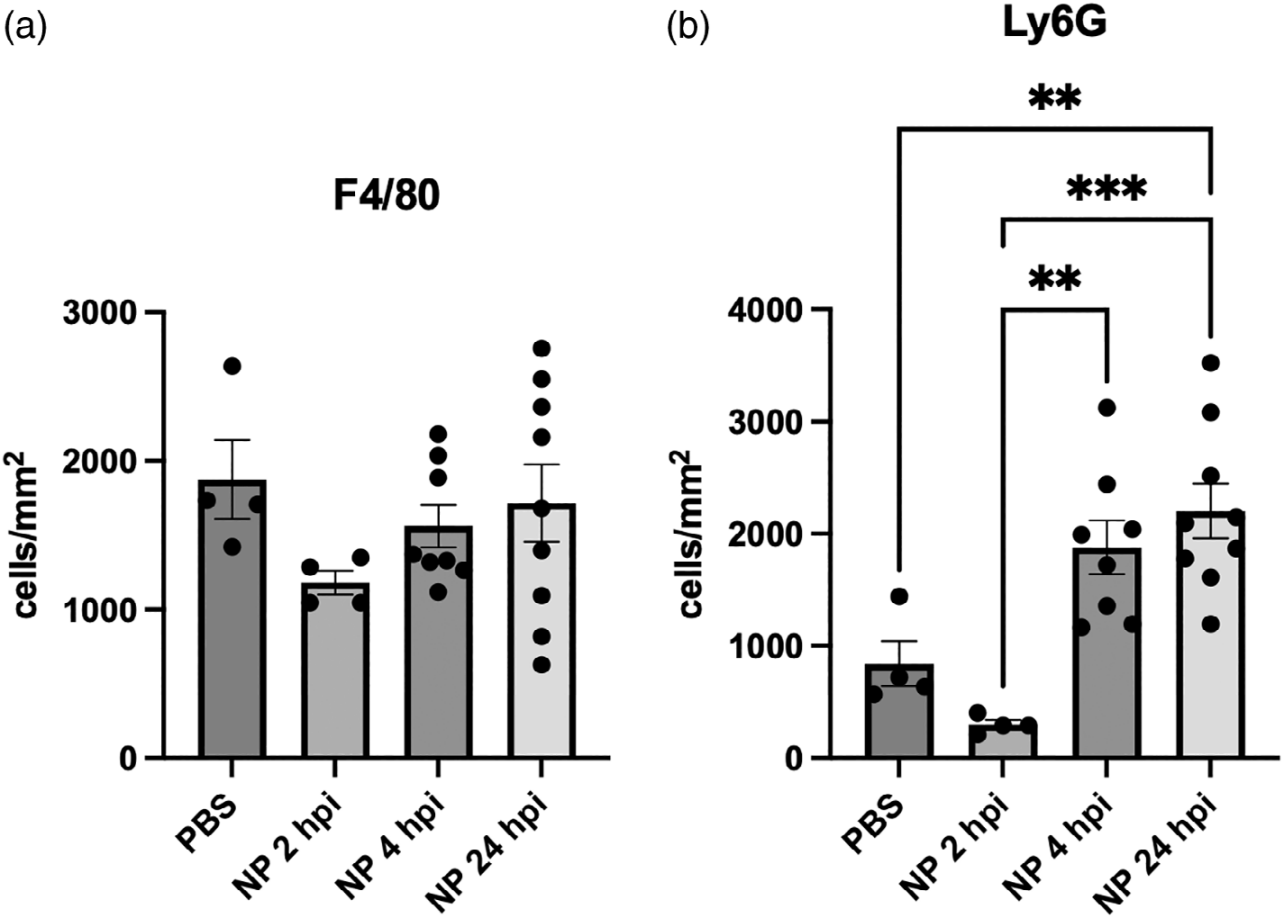
Immune cell infiltration in spinal cord at 7 dpi. Mice received daily injections of NPs or PBS control starting at varying times within the first day of injury through intravenous injection for 1 week. Spinal cords were harvested at 7 dpi, and IHC for macrophages (a) and neutrophils (b) was performed. Data are mean ± SEM. *n* = 4–9 mice per condition. *p < 0.05, **p < 0.01, ***p < 0.001.

We investigated the efficacy of NPs when the initial administration occurs at distinct time points following injury, which may reflect the delays in treatment that may be present in a clinical situation. NPs were initially administered at 2, 4, and 24 hpi, with daily administration until 1 week. The timing of nanoparticle administration remained staggered throughout the entire week to retain administration timing differences between groups. At 7 dpi, spinal cords were harvested, and immune cell infiltration was quantified by immunohistochemistry for F4/80+ macrophages/microglia (Figure 2a) and Ly6G+ neutrophils (Figure 2b). Consistent with our previous studies, NP administration starting within 2 hpi trended toward a decrease in the number of F4/80+ macrophages/microglia and Ly6G+ neutrophils compared to PBS controls. However, these results were not significantly different. While F4/80+ macrophages were significantly lower at 7 dpi in NP 2 hpi vs. PBS mice using flow cytometry in Figure 1c, neutrophil and total immune cell percentages were not significantly lower, which explains the lack of significant difference using IHC quantification. Interestingly, delayed NP injections starting at 4 and 24 h resulted in higher Ly6G+ neutrophil infiltration than the PBS control (NP 24 hpi vs. PBS: *p* = 0.0072) and early NP intervention (NP 24 hpi vs. NP 2 hpi: *p* = 0.0002, NP 4 hpi vs. NP 2 hpi: *p* = 0.0022). This result may be the result of NP administration being too late to affect neutrophil migration to the spinal cord, which typically starts within 3 h of injury.

### 3.3 NP administration enhances neuron sparing in the spinal cord

**FIGURE 3:**
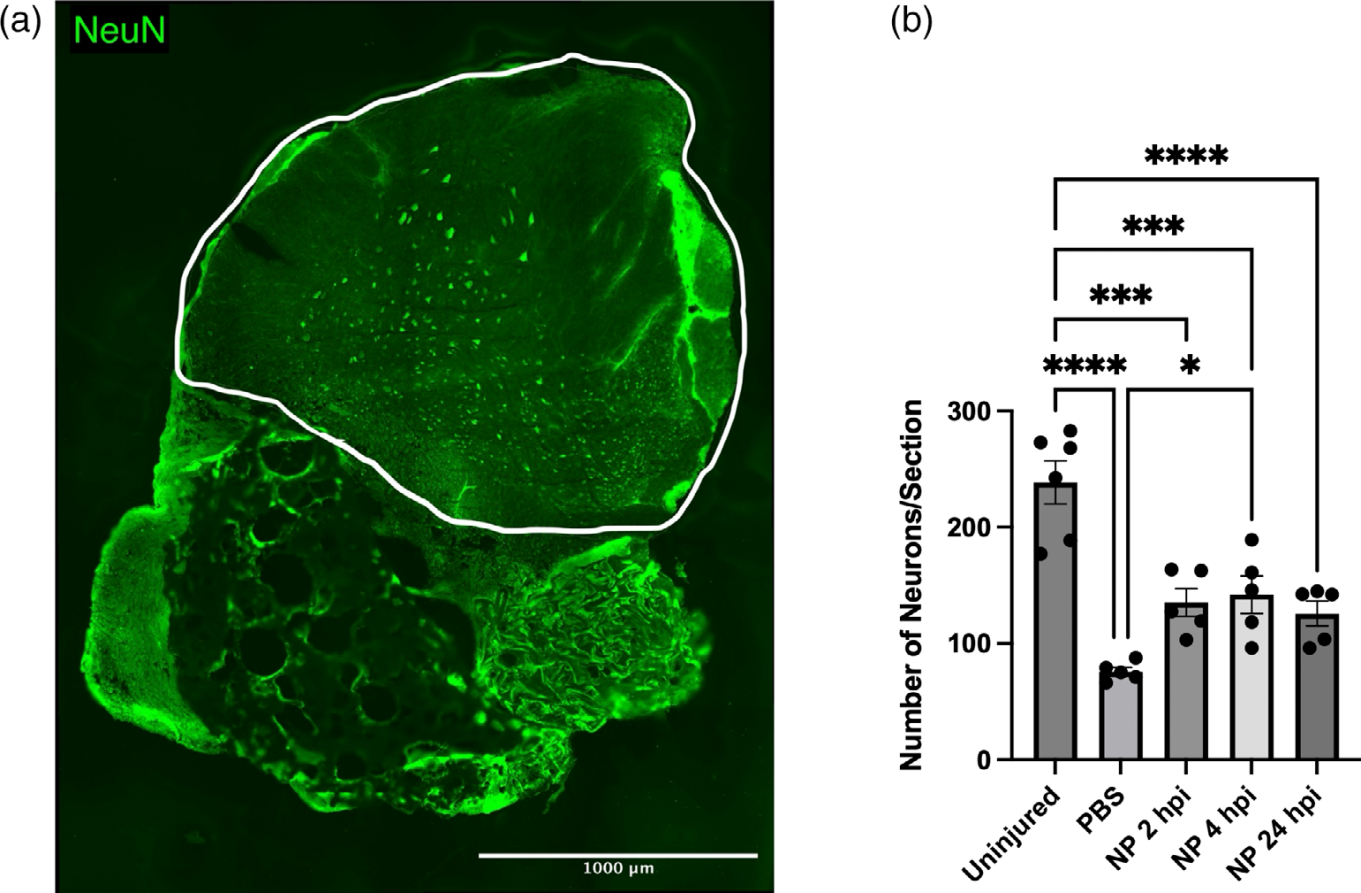
Neuron quantification contralateral to injury at 7 dpi. Mice received daily injections of NPs or PBS control starting at varying times within the first day of injury through intravenous injection for 1 week. Spinal cords were harvested at 7 dpi, and IHC was performed for neurons (a). Neurons contralateral to the C5 hemisection were quantified (b). Data are mean ± SEM. *n* = 5–7 mice per condition. *p < 0.05, **p < 0.001, ****p < 0.0001.

We next hypothesized that NP delivery has the ability to influence neuron sparing within the injured spinal cord. Following spinal cord injury, inflammation and excitotoxicity resulting from the trauma and inflammatory cytokine release can induce widespread neuron death. Immunohistochemistry was performed at 1 wpi for NeuN+ mature neurons within the contralateral side to the injury, where inflammatory cytokine concentration should be highest (Figure 3a, circled in yellow). All injured conditions had fewer neurons than uninjured controls, and NP administration starting at 2 and 4 hpi trended toward increased neuron count compared to the PBS control (*p* = 0.0521 and 0.0145) (Figure 3b).

### 3.4 NMJ sparing is enhanced by early NP administration

**FIGURE 4:**
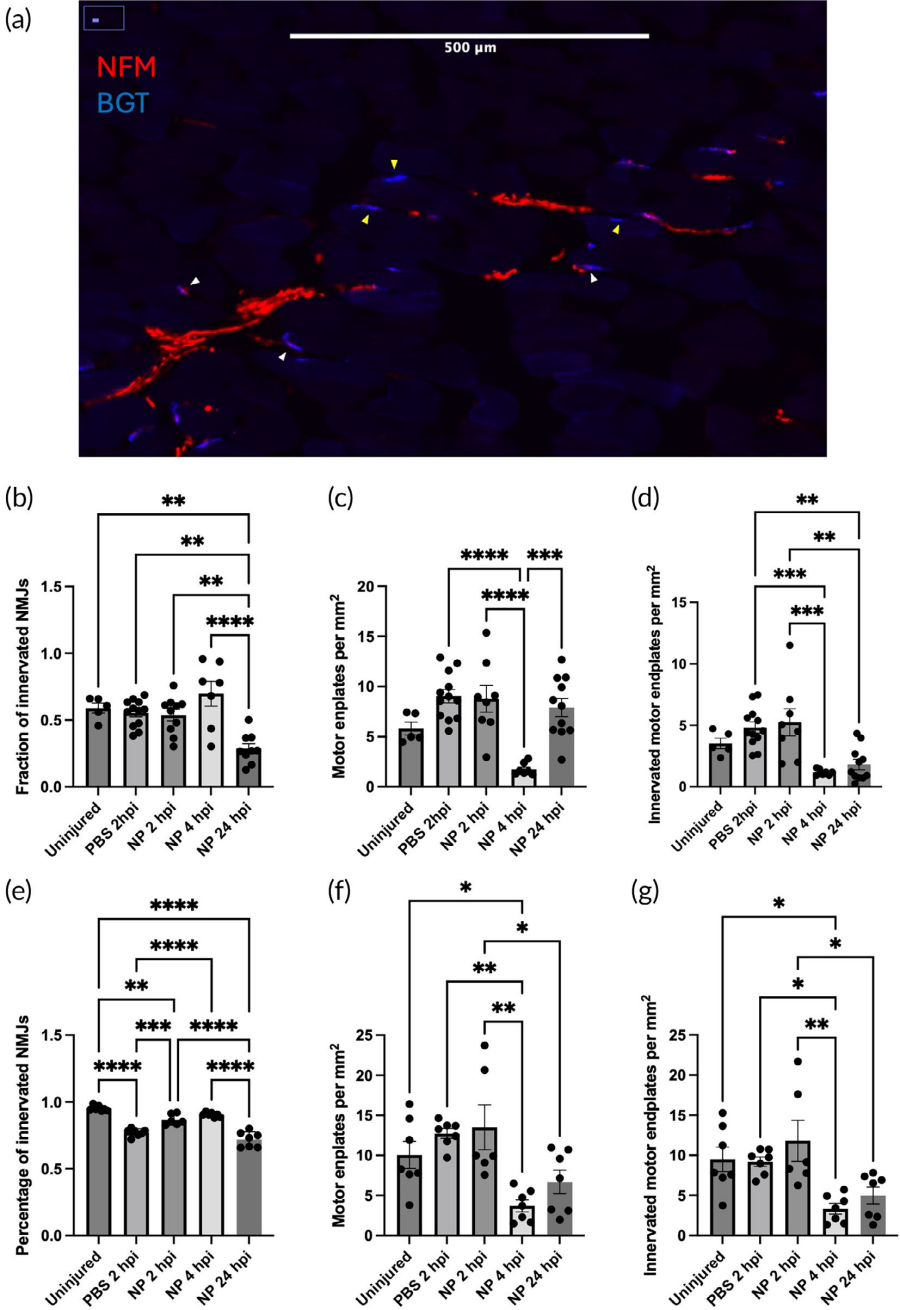
Neuromuscular junction innervation at 7 dpi. Mice received daily injections of NPs or PBS control starting at varying times within the first day of injury through intravenous injection for 1 week. Muscles were harvested at 7 dpi, and IHC was performed for axons (neurofilament medium chain, NFM) and motor endplates (bungarotoxin, BGT). (a) NMJs were quantified from the acromiotrapezius muscle (b–d) and flexor carpi ulnaris and flexor digitorum profundus (e–g). Data are mean ± SEM. *n* = 5–10 mice per condition. *p < 0.05, **p < 0.01, ***p < 0.001, ****p < 0.0001.

We next investigated the correlation between NP administration and the sparing of neural circuits. These circuits were quantified in the muscle of the acromiotrapezius (ATZ) and forearm flexor muscles through IHC for bungarotoxin+ (BGT+) motor endplates and neurofilament 200+ axons (NFM+) (Figure 4a). SCI can cause the death of lower motor neurons, causing the denervation of NMJs. Prolonged loss of synaptic input leads to the disintegration of orphaned motor endplates and muscle atrophy.^29^, ^30^, ^31^, ^32^ The ATZ is innervated from C5 and therefore directly affected by the injury (Figure 4b–d), whereas the flexor muscles are innervated from C6 below the injury (Figure 4e–g).^33^ In the ATZ, NP treatment did not significantly increase sparing of NMJs compared to PBS controls. However, in the flexor muscles, early NP treatment starting at 2 hpi had a significantly greater percentage of innervated motor endplates compared to control (*p* = 0.0004). Delayed NP treatment starting at 4 and 24 hpi led to decreased sparing of motor endplates and innervated NMJs in both the ATZ and flexor muscles. This may be an effect of the increased infiltration of neutrophils and macrophages at 1 wpi in these conditions. Overall, early NP treatment may not be able to spare NMJ innervation that is associated with the hemisection, but it can help spare innervation caudal to the injury site.

### 3.5 Early NP administration increases locomotor functional sparing

**FIGURE 5:**
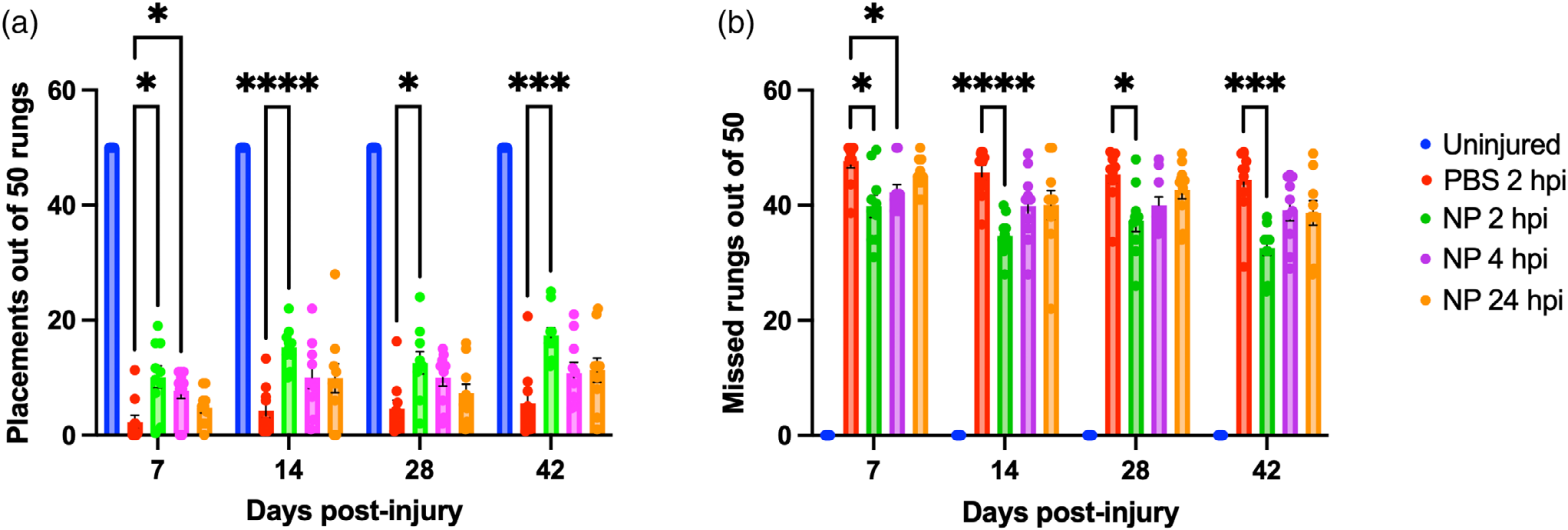
Early NP treatment improves ladder beam performance. The horizontal ladder beam test was performed weekly following injury. The number of correct placements (a) and incorrect placements (b) were counted. Data are mean ± SEM. *n* = 10 per condition. *p < 0.05, ***p < 0.001, ****p < 0.0001.

Mice underwent a horizontal ladder beam test to investigate whether the spared neurons and NMJs in the NP conditions correlated with better locomotor function. At 7 dpi, mice that received NPs within 4 hpi performed significantly better than PBS controls, with more correct placements (Figure 5a) and fewer missed rungs (NP 2 hpi vs. PBS: *p* = 0.0243, NP 4 hpi vs. PBS: *p* = 0.0491) (Figure 5b). Mice that did not receive NPs until 24 hpi performed similarly to mice that only received PBS in the number of misses. After 7 dpi, the motor performance of the NP 2 hpi condition remained steady, but the late NP condition improved over 6 weeks. The performance of the PBS group did not significantly change over the course of the experiment. Collectively, NP administration improved locomotor recovery during the acute and subacute phases of injury if administered within 4 h after SCI.

## 4 DISCUSSION

In these studies, we investigated the use of NPs to reprogram circulating immune cells to enhance regeneration and functional recovery after penetrating spinal cord injury. While contusion and compression injuries are more common, patients with penetrating injuries experience little spontaneous recovery after injury, and there are no clinical therapies designed to reconnect severed spinal cord tracts.^34^, ^35^ SCI leads to the activation of innate immune cells, such as inflammatory monocytes and neutrophils, in the blood and spleen, which can subsequently traffic to the injured spinal cord.^36^, ^37^ This infiltration into the SCI can induce damage secondary to the initial injury, thereby contributing to neuronal cell death, axonal dieback, demyelination, and scar tissue formation.^18^, ^38^ Studies that have attempted to deplete these circulating cells have indicated worsened histological and functional outcomes.^8^ NPs, in contrast, do not function to deplete immune cells and instead reprogram their function. After injury, the activated innate immune cells, which have high phagocytic capacity for scavenging cell debris and foreign materials, can internalize the NPs while in circulation. This internalization of the NPs can alter the trafficking and functional phenotype of the innate cells,^28^ which has led to improved functional recovery.^17^ The immune response following SCI can be highly dynamic, and herein we investigated the timing of NP administration.

NP administration, regardless of the timing for the initial administration, had a different immune cell composition in the spinal cord at 1 week post‐injury. Previous reports had indicated that NPs delivered at 2 hpi decreased the number of inflammatory monocytes and neutrophils at 1 week post‐injury.^17^ Herein, we observed a decreased number of macrophages within the injury for all mice that received nanoparticles. Interestingly, mice that received NPs within 4 h of injury also had fewer neutrophils in the spinal cord, yet mice receiving particles starting at 24 hpi had a greater accumulation of neutrophils at 1 week relative to control. Analyzing responses over time, only NP administration at 2 hpi resulted in a smaller percentage of myeloid cells at 2, 5, and 7 dpi. The immune cell dynamics play an important role in the response that leads to their repair or regeneration.

NP injections within 4 h of injury yielded greater preservation of motor function at 1 wpi relative to later administration, with retention of this increased function through Day 42. This is consistent with human clinical trials of the immunosuppressant methylprednisolone. When patients received a 24‐h long infusion of methylprednisolone within 8 h of injury, mean motor scores improved at 6 and 12 months post‐injury.^39^ However, when methylprednisolone was started later than 8 hpi, there was no motor improvement compared to controls, and this was performed for 24 h in a hospital setting, whereas NPs could be administered by EMTs, and NP administration takes mere minutes. Inflammatory cytokines and reactive oxygen species released by pro‐inflammatory neutrophils and macrophages after SCI are cytotoxic toward neuronal cells.^40^, ^41^ IL‐1α and TNFα in the injury microenvironment induce increased trafficking of glutamate receptors to neuron cell membranes and thereby cause increased excitotoxicity.^42^, ^43^ NP administration is able to alter the immune cell accumulation and polarization normally seen following SCI and contribute to the increased function by increasing sparing of neurons and motor circuits. Previous studies with NP injection had been performed with a thoracic injury, and BMS scores for NP treated mice were increased relative to control mice by Day 7.^17^ For the study herein that had a cervical injury, an increased functional response was again observed as early as Day 7 for mice receiving particles at 2 hpi relative to control. At 1 wpi, all NP‐treated mice, regardless of the timing of initial NP administration, had a larger number of surviving neurons contralateral to the injury than mice that did not receive NPs. Mice that received NPs starting at 24 hpi had fewer intact NMJs than even control mice receiving PBS injections at 2 hpi. While we used both male and female mice in equal numbers in this study, the number of animals used was not sufficiently powered to study sex‐based differences in immune response and neural sparing. This may be significant due to male and female mice exhibiting different immune responses following injury, with male mice exhibiting worse microglia accumulation in the injury and worse neuron sparing as a result.^44^, ^45^ Our results suggest that even though neuron survival is not dependent upon the time of first NP administration, early administration of NPs may maintain neuronal connections more effectively than later administration.

Mice with a spinal cord injury that received NPs at 24 hpi initially had motor performance similar to control mice, yet their motor performance improved over time. While an unchecked immune response after SCI can lead to neuron cell death and axonal dieback, an attenuated inflammatory response can have reduced damage, such as demyelination of nerve fibers without axonal death, which leads to decreases in their conductive potential and functional deficits.^46^, ^47^ After the acute phase of injury, the surviving nerve fibers may have conduction recover partially or completely.^47^ For the mice receiving particles later, we hypothesize that the initial deficit and gradual improvement may be associated with recovery from the conduction block. These mice had greater neuronal and NMJ sparing than mice that receive NPs at later times; thus, the recovery in function that was observed during subacute and chronic phases may reflect partial recovery in conduction in the spared motor circuits. Within the mice receiving NPs at 24 hpi, we anticipate that the recovery of function was associated with this recovery in the conduction of spared circuits. However, the initial immune response decreased the extent of spared NMJs relative to NPs delivered at earlier times, and thus the extent of recovery was more modest.

In conclusion, this report demonstrates that NP treatment can modulate the inflammatory microenvironment after SCI, and that NP administration within 24 h is associated with a greater recovery of function relative to control. While the numbers of infiltrating immune cells are similar in NP‐treated and control mice 2 days post‐injury, fewer cells were present by the end of the first week post‐injury. However, these results may be valid only for penetrating injuries rather than the more common contusion, which has differing immune cell dynamics and inflammation spread following injury, and therefore need to be verified in this model before generalizing our results to all types of SCI.^48^, ^49^ Additionally, mice were assigned a condition using a random number generator, so there may be human bias in assigning mice to the treatment group. Further studies are also needed to investigate the direct effects of the NPs on trafficking of myeloid cell populations to the injury versus indirect effects on myeloid cell trafficking due to passive accumulation of NPs in the injury. In addition to altering the immune microenvironment in the spinal cord, NP administration leads to increased sparing of neurons. Early administration of NPs is able to substantially attenuate the early functional deficits. Administration of NPs at late time points is associated with early functional deficits, yet these functional deficits improve with time, with greater levels of improvement with earlier timing of NP administration. These NPs may provide a therapeutic option for patients with SCI to limit secondary damage, as they are formed from a material that has been approved by the FDA for use in other indications and is stable and could be available off‐the‐shelf.

## AUTHOR CONTRIBUTIONS

**Sarah E. Hocevar:** Conceptualization; writing – review and editing; methodology; investigation; writing – original draft; formal analysis. **Brian C. Ross:** Writing – review and editing; investigation; formal analysis. **Yinghao Wang:** Investigation. **Cecelia R. Crowther:** Investigation. **Samantha R. Schwartz:** Writing – review and editing; investigation. **Brain J. Cummings:** Writing – review and editing. **Aileen J. Anderson:** Writing – review and editing. **Lonnie D. Shea:** Conceptualization; writing – review and editing; writing – original draft; supervision; methodology; funding acquisition.

## CONFLICT OF INTEREST STATEMENT

We declare a conflict of interest for Dr. Shea, who consults and has financial interests in Cour Pharmaceutical, which has licensed the nanoparticle technology and aims to commercialize the nanoparticles. Cour did not directly support the work submitted herein.

## Data Availability

The data that support the findings of this study are available from the corresponding author upon reasonable request.
